# Higher hemoglobin levels are associated with better physical performance among older adults without anemia: a longitudinal analysis

**DOI:** 10.1186/s12877-022-02937-4

**Published:** 2022-03-21

**Authors:** Ligiana Pires Corona, Flavia Cristina Drumond Andrade, Tiago da Silva Alexandre, Tábatta Renata Pereira de Brito, Daniella Pires Nunes, Yeda Aparecida de Oliveira Duarte

**Affiliations:** 1 Faculty of Applied Sciences, University of Campinas (UNICAMP), R. Pedro Zaccaria, 1300 – 13484-350, Limeira, SP Brazil; 2 School of Social Work, University of Illinois at Urbana-Champaign, 1010 W. Nevada St, Urbana, IL 61801 United States of America; 3 Gerontology Department, Federal University of Sao Carlos, Rodovia Washington Luís, km 235, SP-310, São Carlos, SP Brazil; 4 Federal University of Alfenas, Rua Gabriel Monteiro da Silva, 976, Alfenas, MG Brazil; 5 Medical-surgical Nursing Area, Faculty of Nursing, University of Campinas (UNICAMP), R. Tessália Vieira de Camargo, 126, Campinas, SP Brazil; 6 Department of Medical-Surgical Nursing, School of Nursing, University of São Paulo, Av. Dr. Enéas de Carvalho Aguiar, 419, São Paulo, SP Brazil

**Keywords:** Hemoglobin, Physical performance, Older adults, SABE study, Brazil

## Abstract

**Background:**

Anemia is the most common hematological abnormality among older adults, and it is associated with decreased physical performance. But the role of hemoglobin in the absence of anemia remains unclear. Thus, this study aimed to assess the impact of hemoglobin levels on physical performance in Brazilian older adults without anemia.

**Methods:**

The study is longitudinal in that it relies on two waves of the Saúde, Bem-Estar e Envelhecimento (SABE; Health, Well-being, and Aging) study: 2010 and 2015-2016. Mixed-effects linear regression was used to determine the effects of the hemoglobin concentrations on the Short Physical Performance Battery-SPPB over time among the 1,023 who had complete data and did not have anemia in 2010. In the follow-up, there were 567 without anemia.

**Results:**

In analyses adjusted for age, education, comorbidities, body mass index, and physical inactivity, we found a differential association between hemoglobin concentration and SBBP by sex, with a positive interaction (β Hb*female= 0.20, 95% CI 0.04,0.37). At lower levels of hemoglobin, women have lower levels of SPPB than men, but at higher levels of hemoglobin concentration, there are no sex differences in physical performance. In addition, higher age was negatively associated with SPPB levels and cardiometabolic diseases, other diseases, and physical inactivity. Education was positively associated with physical performance.

**Conclusion:**

Our study demonstrates that higher hemoglobin levels were associated with better physical performance among older adults without anemia in Brazil. However, there were sex differences in this association. This finding is important because, in clinical practice, most health professionals focus on the World Health Organization definition of anemia. Our study suggests the importance of hemoglobin levels among older adults, even those without anemia, and highlights sex differences.

**Supplementary Information:**

The online version contains supplementary material available at 10.1186/s12877-022-02937-4.

## Background

It is well-known that anemia, the most common hematological abnormality among older adults, is a major health problem associated with decreased physical performance, reduced mobility, increased functional dependence, and higher mortality [1–6]. However, there is some debate whether the World Health Organization (WHO) criteria to define anemia are adequate for older adults. Previous studies found disability and mortality risk gradients even within the WHO normal hemoglobin (Hb) range, suggesting that Hb levels higher than current cutoffs for anemia treatment might offer a clinical advantage [4, 7, 8]. Therefore, it is important to explore more broadly the impact of Hb levels, rather than anemia thresholds, on physical performance.

A limitation in the past studies is that most that analyzed the association between Hb levels on physical function are cross-sectional. Those that have used longitudinal data have focused on the effect of baseline Hb levels on physical function [9–11]. Chaves et al. [1] conducted a cross-sectional study with older women from the Women’s Health and Aging Studies I and II, which found that mobility difficulty was greater among those participants with lower Hb levels. A study conducted in Australian men described that each 1g/dL increase in Hb was strongly associated with a reduced risk of slow walking speed, poor grip strength, inability to perform chair stands, activities of daily living (ADL), and instrumental activities of daily living (IADL) disabilities [12]. Tseng et al. (2021) found that slower gait speed was significantly associated with lower Hb levels in Taiwanese older adults [11]. Marzban et al. (2021) also showed a short review in their paper with several other studies [10], but none discussed this effect in individuals without anemia.

There are few studies that have considered Hb changes, but they have produced conflicting results, and some have analyzed narrow populations. For example, Zakai and colleagues (2005) reported that lower baseline Hb levels and incident anemia were associated with lower gait speeds among women at follow-up even though the change in Hb levels over time was not statistically significant [4]. Hirani et al. (2016), in a sample of Australian men, showed that for every 1 g/dL increase in Hb, there was a significant reduction in risk of sarcopenia, slow walking speed, poor grip strength, inability to perform chair stands, and problems with ADLs and IADLs [12]. Yoshimura et al. (2021) showed that the change in Hb levels was positively associated with the Functional Independence Measure - motor efficacy. Still, the sample was restricted to stroke patients with anemia who had been hospitalized [13].

Cross-sectional studies in low and middle-income countries (LMIC) have explored the associations between hemoglobin or anemia and several outcomes, such as higher health services utilization, disability, frailty, and mortality [6, 8, 14–16]. Still, no longitudinal studies have been used to examine the role of Hb on physical performance. Conducting these studies in LMIC is even more important when evidence reveals global inequalities in the prevalence of anemia (9% in developed and 43% in developing countries). Socioeconomic disparities reflect differences between them, being the poorest and least educated more exposed to risk factors for anemia [17]. Thus, considering the association between anemia and physical performance and social inequality has also been associated with lower physical performance in older ages [18], studies in LMIC can help guide public health actions.

Some cross-sectional studies also showed that the association between Hb and functionality might differ between men and women. Payne et al. (2018) analyzed data from a population-based survey of rural South African men and women aged 40 and over. The authors did the analyses separately by sex, given that women have lower hemoglobin and muscle strength levels than men [9]. They found that Hb concentration was positively associated with grip strength in women but not in men and did not observe associations between Hb and walking speed [9]. In a study based in Japan, Sawada et al. (2021) analyzed data separately by sex and showed low Hb was associated with worse scores in IADLs and cognition in women but not in men [19]. A study conducted with Iranian older adults found that Hb concentration was negatively associated with walking speed in full sample [10]. However, when the analyses were performed separately by gender, Hb concentration association was no longer associated with usual gate speed in both men and women in fully adjusted models [10].

As pointed out before, men and women tend to have different Hb concentration levels and differ on physical performance measures. These differences may be due to age-related changes in testosterone in man, and higher morbidity in women in contrast with the cessation of menstrual blood loss in older ages [9, 10, 19]. However, the differences in physical function and hemoglobin levels according to sex are not fully explored in the literature.

Thus, this study aimed to address some of the limitations of past studies. To do so, we assess the impact of Hb concentration on physical performance in Brazilian older adults without anemia and examine whether these associations differ by sex using longitudinal data.

## Methods

### Design and participants

Data from the Saúde, Bem-Estar e Envelhecimento (SABE; Health, Well-being, and Aging), a multiple-cohort study of Brazilian older adults that began in 2000, are used in this study. SABE is based on a probabilistic sample of adults aged ≥60 years residing in the city of São Paulo (*n*=2,143). Follow-ups were conducted in 2006, 2010, and 2015-2016. A representative sample of older adults aged 60 to 64 years was added to the study in each new wave. These additions increased the sample size and ensured the representativeness of the population 60 years and older. Details on the methodology of the study have been published previously [6, 8]. Participation was voluntary, and a signed informed consent form was obtained from all participants in each wave. All of the procedures followed the ethical standards of the institutional and national research committee and the 1964 Helsinki declaration.

**Fig. 1:**
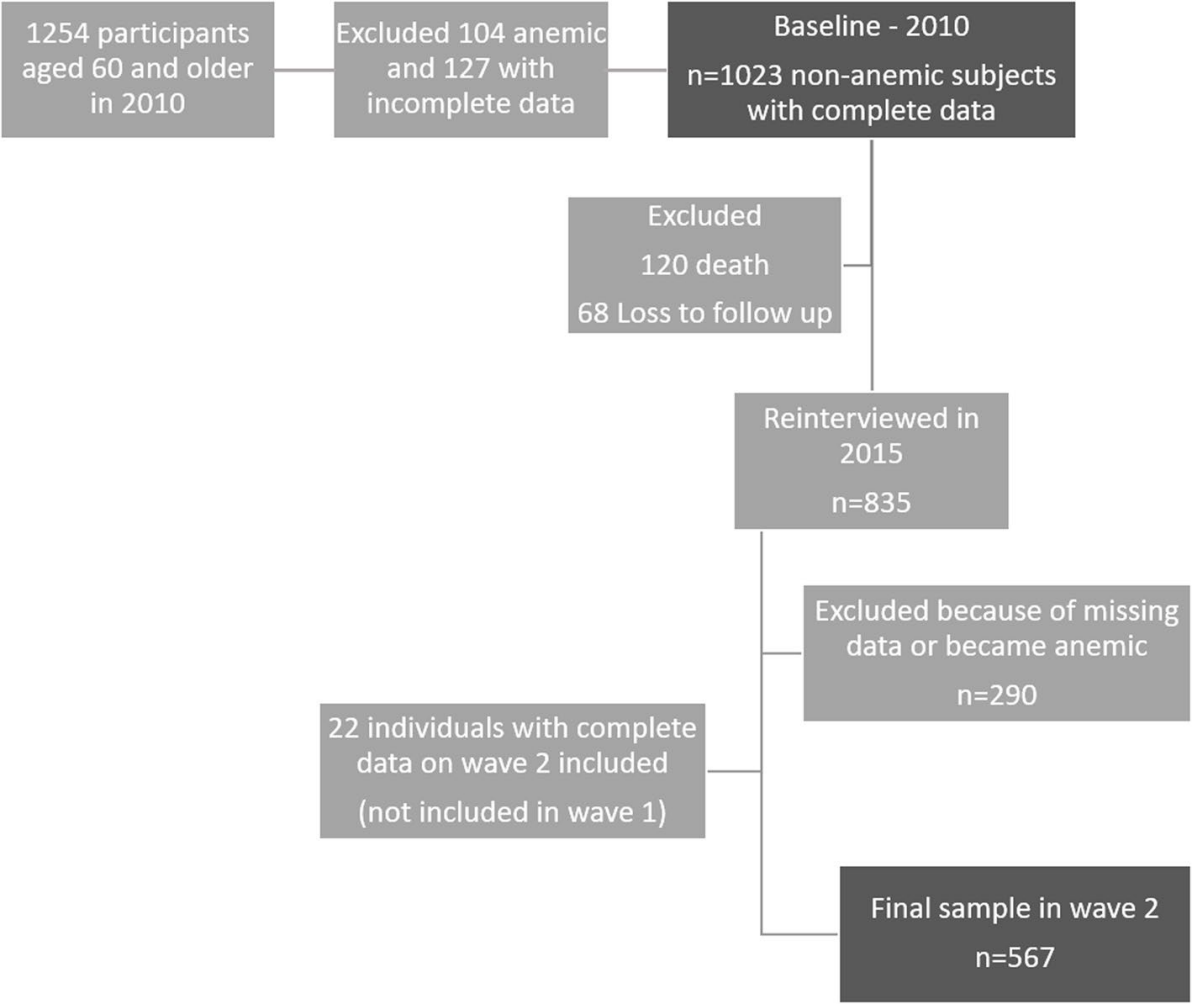
Flowchart of the study sample, SABE Study, São Paulo, Brazil (from 2010 baseline to the follow-up in 2015)

For this study, we focus on the last two waves as blood samples were first collected in 2010. In 2010, 1,254 participants were aged 60 and older, but 127 had incomplete data on blood parameters or selected variables. Another 104 were anemic. Therefore, the 2010 sample is restricted to 1,023 individuals. In 2015, 835 individuals of these individuals were reinterviewed. Among these, 290 either had missing data (*n*=235) or developed anemia (*n*=55). Another 22 participants were either added in the second wave (*n*=10) or missing only in the baseline (*n*=12), leaving 567 in the follow-up. Fig. 1 shows the flowchart of the study sample, and supplementary table 1 shows a comparison between those with complete data in the second wave and the 478 excluded during the follow up period.

### Measures and procedures

Data collection included face-to-face interviews using a standardized questionnaire, measurement of anthropometric data and physical performance, and collection of physiological specimens of blood and urine.

The outcome measure, physical performance, was assessed in both waves using the Short Physical Performance Battery - SPPB [20, 21]. The total SPPB score is the sum of the scores on the three tests and ranges from 0 to 12: standing balance (the ability to maintain feet side-by-side, semi-tandem, and tandem positions for 10 seconds each), walking speed (for 3 m at their usual pace), and ability to rise from a chair (stand up from a sitting position once with arms folded at the chest).

Venous blood was collected at both waves. Hemoglobin concentration (g/dL) was analyzed at the Hospital of the Medical School laboratory at the University of São Paulo using an ADVIA 120 system (Siemens Healthcare Diagnostics, Germany). Anemia was defined using the WHO cutoffs – hemoglobin <12 g/dL for women and <13 g/dL for men [22], and anemic individuals were excluded from the analysis.

Covariates were measured in the baseline. We included age, education (in years of schooling), health status, body mass index (BMI), and physical inactivity. Health status was assessed based on self-reported chronic conditions diagnosed by a physician. We combined these conditions into two groups: 1) the number of cardiometabolic diseases (diabetes, hypertension, stroke, and cardiovascular disease) because of their interaction with anemia in physical function [23], and 2) the number of other self-reported chronic conditions (cancer, chronic pulmonary disease, osteoarthritis, and osteoporosis). BMI was calculated by dividing weight (in kilograms) by height (in meters) squared (kg/m^2^). Weight was measured using a calibrated scale, and height was measured using a stadiometer fixed to a plain wall, both with the individuals barefoot and wearing light clothing. Physical activity level was measured using the International Physical Activity Questionnaire (validated in Brazil) [24]. Individuals were classified as physically inactive if they reported less than 150 minutes of moderate activity or less than 75 minutes of vigorous activity per week [25].

### Statistical analysis

Descriptive statistics (means and standard errors) were generated to assess the differences in physical performance and selected variables by sex in 2010, except for physical inactivity (evaluated using chi-squared test). The sex comparisons were conducted using Student’s t-test for continuous variables. To test the association between hemoglobin concentration and SPPB scores, we conducted a repeated mixed-effects linear regression with robust standard errors [26]. Repeated mixed-effects regressions handle nested data inherent to repeated observations within individuals to allow for an unequal number of observations across individuals. SPPB score and hemoglobin concentration were treated as continuous variables and considered time-varying since the measures were collected in both waves.

Random effects for the intercept were included to allow individuals to vary in the initial level of physical performance. We present four hierarchical models, including the covariates in blocks as follows: Model 1 – time, age, Hb, and sex (and the interaction between Hb and female sex); Model 2 – Model 1 + education (in years of schooling); Modelo 3 – Model 2 + health conditions (number of cardiometabolic diseases, the total number of other chronic conditions); Model 4 – model 3 + BMI and physical inactivity (final model). We present the regression coefficients, confidence intervals, and *p*-values. To assess the fit of nested models, we used Bayesian (BIC) and Akaike information criteria (AIC). Likelihood-ratio tests are not appropriate for the goodness of fit comparisons as we use robust standard errors. Model diagnosis showed the assumption of normally distributed residuals was met. Also, predicted values were close to the observed ones. Homocedasticity of the errors seems to be met.

To facilitate the interpretation of regression results, particularly of the sex interaction with Hb levels, we examined the linear predictions obtained with the “margins” command. We use the “marginsplot” command to graph the influence of the hemoglobin concentration on the SPPB score. All data analyses were conducted using the statistical software Stata/SE 16.1.

## Results

Table 1 displays some selected characteristics of participants at the baseline. Most of the evaluated population was female. Women were significantly less educated, had more health conditions, higher BMI values, lower Hb, and lower SPPB scores in 2010. There were no sex differences in physical inactivity.

**Table 1.** Mean and standard errors of selected characteristics of older adults (≥ 60 years old) according to sex in the baseline. SABE Study. São Paulo, Brazil, 2010.

Characteristics	Total (*n* = 1023)	Men (*n* = 355)	Women (*n* = 668)	*p*
Age	71.56 (0.28)	71.38 (0.47)	71.65 (0.35)	0.648
Education (in years)	4.85 (0.13)	5.29 (0.22)	4.62 (0.16)	0.015
Number of cardiometabolic conditions	1.20 (0.03)	1.09 (0.05)	1.26 (0.04)	0.006
Number of other chronic conditions	0.71 (0.02)	0.36 (0.03)	0.89 (0.03)	<0.001
BMI (kg/m^2^)	28.21 (0.16)	26.89 (0.22)	28.91 (0.22)	<0.001
Physical inactivity (%)	47.21	48.30	46.62	0.569
Hemoglobin concentration (g/dL)	14.30 (0.03)	15.06 (0.06)	13.90 (0.34)	<0.001
SPPB score	8.89 (0.07)	9.03 (0.11)	8.67 (0.09)	<0.001

*SE* Standard Error, *95% CI* 95% Confidence Interval, *SPPB* Short Physical Performance Battery, *BMI* body mass index

**Fig. 2:**
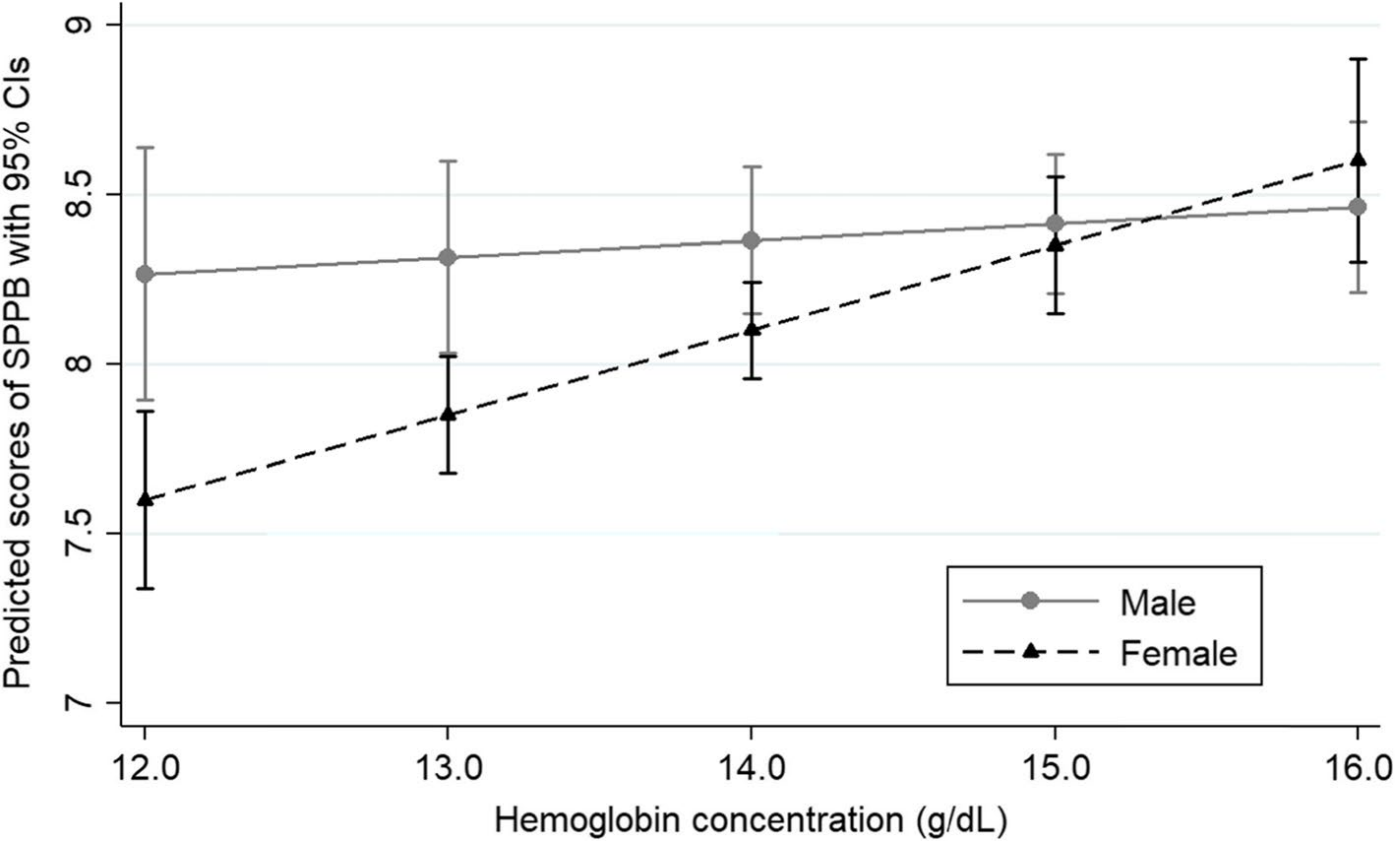
Predicted scores of Short Physical Performance Battery (SPPB) for older men and women in Brazil. SABE Study. São Paulo, Brazil, 2010-2015

Table 2 shows the results of the adjusted mixed-effects linear models. In all models, there was a significant interaction between sex and hemoglobin levels. Results based on Model 4 show that women have, on average, lower levels of SPPB than men (β _female_= -3.08, 95% CI -5.45, -0.70). Still, there is a positive interaction (β Hb*female= 0.20, 95% CI 0.04,0.37), indicating a differential association between hemoglobin concentration and SBBP by sex. The interaction can be better visualized in Fig. 2, which shows that at lower levels of hemoglobin, women have lower levels of SPPB than men, but each one-unit increase in hemoglobin among women is associated with higher gains in SPPB levels. At higher levels of hemoglobin concentration, there are no sex differences in physical performance. In addition, regression results indicate reductions in mean levels of SPPB over time across waves. Higher age was negatively associated with SPPB levels and cardiometabolic diseases, other diseases, and physical inactivity. Education was positively associated with physical performance.

**Table 2.** Repeated mixed-effects linear models for longitudinal changes in physical function (SPPB score) as a function of hemoglobin changes over five years in Brazilian older adults. SABE Study. São Paulo, Brazil, 2010-2015.

	Model		Model 2		Model 3		Model 4	
	β	95% CI	β	95% CI	β	95% CI	β	95% CI
*Fixed*								
Year	-1.95***	-2.14,-1.77	-1.96***	-2.14,-1.77	-1.97***	-2.15,-1.79	-1.93***	-2.11,-1.75
Age	-0.13***	-0.14,-0.11	-0.12***	-0.13,-0.10	-0.11***	-0.12,-0.10	-0.11***	-0.12,-0.10
Hemoglobin concentration (g/dL)	0.1	-0.04,0.24	0.11	-0.03,0.25	0.06	-0.07,0.19	0.05	-0.07,0.17
Sex (female)	-3.32*	-6.02,-0.62	-3.02*	-5.71,-0.33	-3.22*	-5.75,-0.69	-3.08*	-5.45,-0.70
Hemoglobin*female interaction	0.21*	0.02,0.39	0.19*	0.00,0.38	0.21*	0.04,0.39	0.20*	0.04,0.37
Education			0.10***	0.07,0.13	0.09***	0.07,0.12	0.09***	0.06,0.11
Cardiometabolic conditions					-0.49***	-0.62,-0.36	-0.41***	-0.53,-0.28
Other chronic conditions					-0.22**	-0.37,-0.06	-0.23**	-0.38,-0.07
BMI (kg/m^2^)							-0.02	-0.04,0.01
Physical inactivity							-0.73***	-0.96,-0.50
Constant	16.92***	14.43,19.41	15.37***	12.87,17.87	16.35***	14.02,18.69	17.25***	14.98,19.53
*Random*								
Intercept	1.68	1.53,1.83	1.63	1.49,1.78	1.55	1.41,1.71	1.44	1.30,1.58
Residual	1.63	1.52,1.75	1.63	1.52,1.75	1.63	1.52,1.75	1.61	1.51,1.73
BIC	7993.785			7955.379		7900.628		7630.963
AIC	7949.865			7905.975		7840.244		7702.172

*95% CI* 95% Confidence Interval, *BMI* body mass index, *AIC* Akaike Information Criterion, *BIC* Bayesian Information Criterion. Model 1 – time, age, Hb and sex (and the interaction between Hb and female sex); Model 2 – Model 1 + education (in years of schooling); Modelo 3 – Model 2 + health conditions (number of cardiometabolic diseases, total number of other chronic conditions); Model 4 – model 3 + BMI and physical inactivity (final model).

^*^p <0.05; **p < 0.01; ***p < 0.001

## Discussion

In our results, Hb levels were positively associated with SPPB in older adults without anemia – those with higher Hb levels presented better physical performance. To our knowledge, this is the first study to analyze this association in older adults without anemia in low and middle-income countries. Our results confirm previous findings by Hirani and colleagues’ in which increases in Hb levels are associated with better physical performance indicators, but their results were limited to men in Australia. In our study, we show a significant interaction between hemoglobin concentration and sex. At lower levels of hemoglobin concentration, older Brazilian women without anemia have lower levels of SPPB than men. Still, at higher concentrations of hemoglobin, there are no sex differences in physical performance.

Considering sex differences in the analysis is important because the relationship between Hb and physical function in men and women is not fully yet understood. Sawada et al. (2021) found an association between Hb level and scores on IADL and MMSE in women, justifying their results as "sex morbidity-mortality paradox", a contradiction of higher female morbidity but higher male mortality as first described by Verbrugge [27]. They also suggested that sex differences were associated with body composition in men and worse nutritional status in women [19]. Payne et al. (2018) described a positive association between Hb and grip strength in women but not in men. They mentioned that the prevalence of anemia in their study increased with age among men only. They found that Hb concentrations in the youngest group of healthy women were lower than those seen in older groups, which may reflect the impact of menstrual blood loss in younger women. So, the decrease in Hb levels in women may be patological and could explain the clearer effect in physical function. They also pointed that that reasons for the difference in this relationship between men and women may explained by age-related changes in testosterone [9].

In contrast, Marzban et al. (2021) found a positive association between the hand grip and Hb, consistent in all participants and sex-stratified analysis, even in fully adjusted models. The authors discussed that women have more responsibility in some cultures than men in doing housework such as cooking, cleaning, washing dishes, taking care of (grand) children, and their husbands. Therefore, they could maintain upper limb muscle strength. Besides, older men have a higher chance of losing muscle mass with increasing age than older women. Decreases in physiological factors (e.g., insulin-like growth factor-1 and testosterone) and social factors such as work retirement and loss of social roles might dramatically decrease muscle strength in older men. Also, a negative association between Hb and walking speed was reported when considering the full sample. Still, in sex-stratified analysis, the association decreased in both men and women, being no longer significant in fully adjusted models. They discuss the possible role of education, culture, and body composition to explain the lower significance in women [10].

Previous authors have already suggested that the WHO criteria for defining anemia in adults may not be adequate for older populations. These optimal Hb cutoffs for clinical decision-making were defined mainly based on statistical distribution considerations using apparently healthy people, rather than being developed through considerations of inflammation, the high prevalence of chronic conditions in older adults, and the physiological reduction in Hb concentration in the oldest old, factors that would shift the Hb distribution [1, 28–30].

One cause of the association between Hb and physical performance is that Hb is responsible for tissue oxygenation, and lower Hb values can lead to local hypoxia in skeletal muscle and reduced muscle function [11]. This effect is usually described in people with anemia. However, we found that lower Hb levels, even without anemia, can lead to lower physical function. This finding has not been underexplored in the literature. Zakai et al. [4] suggested that functional decline may occur in close temporal association with hemoglobin decline, reinforcing that analyzing Hb levels may be more informative than only diagnosing anemia, as we showed here.

Steensma and Tefferi (2007) discussed that formal definitions of anemia do not always address the complex relationship between Hb level and health outcomes. Many factors can affect a healthy person’s Hb value, including ethnic background, altitude of residence, smoking status, and physiologic fluctuations of plasma volume. Hence, the interpretation of blood count results remains the responsibility of the ordering physician, who should also refer to a patient’s baseline Hb level when a previous measurement is available [28]. In the same study, Steensma and Tefferi point out that a growing body of medical literature supports a “low-normal” Hb level associated with a broad range of poorer health-related outcomes. For example, a previous cross-sectional study using data from the 2010 wave of SABE study showed that at a concentration of 12 g/dL, the probability of mobility difficulty was 9.1%. But at higher levels, the probability of mobility decreased by 7.4% at 13 g/dL and 6.1% at 14 g/dL [8]. Furthermore, the association was consistent in both men and women [8].

Another important aspect of our study is the specificity of the relationship between Hb concentrations and physical function, which was consistent even after adjusting for age, years of education, number of chronic cardiometabolic conditions, number of other chronic diseases, and BMI an independent association. Those covariates included in our analyses were largely discussed in the literature as risk factors for worse physical function and disability. Some functional decline is expected with advanced age, even without disease, but this decline is slow and gradual [31]. Some authors point out that other factors are determinants for this decline, such as education [8, 32, 33]. It is also well known that physical function decline is higher with chronic diseases [34, 35]. Our analysis also opted to consider cardiometabolic conditions separately because it was already discussed that those conditions have an important interaction with anemia in physical function [13, 23]. We also adjusted for higher BMI, which several publications have indicated as a risk factor for poor physical performance, mainly in walking and chair-stand tests [31, 36–38], and for physical inactivity, one of the main risk factors described in the literature for disability in older ages [25, 39].

Interpretation of our results should consider some limitations. First, as with any other aging cohort, the loss to follow-up and death during the period is considerable, so it is important to assume a possible survival bias. In present study, those lost to follow-up or had died were older, less educated, had more cardiometabolic conditions, lower BMI, Hb and SPPB scores than those who were included in the second wave, as shown in supplementary table 1. Another study based on SABE data shows that anemia predicts mortality among participants [6]; lower hemoglobin values among the non-anemic as well. This suggests that our study underestimates the effects of Hb on physical performance. Another limitation is that the time between the two measurements (five years) is considerably long, which could mask shorter fluctuations. Finally, we are aware that literature has described a U or J shape relationship between Hb levels and severe outcomes, recommending using other iron markers such as hematocrit or iron status to complement the evaluation of iron metabolism [4]. Nonetheless, Patel (2008) points out that the higher mortality associated with higher hemoglobin levels might reflect unmeasured pulmonary disease and/or inadequate adjustment for smoking history [40]. In this paper, we found that higher levels of physical performance at higher Hb concentration levels. Besides, in clinical practice, Hb is the primary marker for anemia and iron status, especially in developing countries, where iron concentrations dosage can be expensive, so it is not part of a routine evaluation in primary care, is usually evaluated only when it is necessary a differential or confirmative diagnosis. So, we believe Hb is an easy and inexpensive marker that should continue to be used in any clinical setting.

But our study has several strengths. First, it is the first study with a large representative sample of community-dwelling older adults in an LMIC, where a nutritional transition is still ongoing. The causes and consequences of lower levels of Hb may represent a heavy burden in health services. Also, our analyses considered the levels of Hb over time in physical function, which is less common as even longitudinal studies typically consider Hb levels only at the baseline. We also showed an interaction effect between sex and Hb, which is important to target health care actions specifically for men and women. Most importantly, we showed that the effects of Hb levels are consistent in non-anemic individuals, which may alert health professionals to the importance of evaluating changes in Hb levels in all older adults, even without the formal diagnosis of anemia.

## Conclusions

In conclusion, our study demonstrates that non-anemic older adults with higher hemoglobin levels presented better physical performance, with an interaction effect between sex and hemoglobin concentration. This result is important because, in clinical practice, most health professionals do not stay alert if Hb level drops but does not reach the WHO-defined anemia threshold. We raise the possibility that therapeutic interventions (such as improvement in nutritional intake, treatment of possible causes of lower levels of Hb, treatment of chronic conditions that may be impacting Hb levels, or pharmacological approaches to correct iron deficiency) may be taken with reductions of Hb levels, even before anemia is diagnosed, to maintain physical function, especially in older women.

## Supplementary Information

**Additional file 1. **

## Data Availability

The datasets on which the conclusions of this manuscript rely are not available publicly. The datasets used and/or analyzed during the current study are available from YAOD, PI of SABE Study (yedaenf@usp.br), on reasonable request.

## Abreviations

ADL: Activities of Daily Living
AIC: Akaike Information Criterion
BIC: Bayesian Information Criterion
BMI: body mass index
CI: Confidence Interval
Hb: hemoglobin
IADL: Instrumental Activities of Daily Living
LMIC: low and middle-income countries
SABE Study: Saúde, Bem-Estar e Envelhecimento (Health, Well-being, and Aging Study)
SE: Standard Error
SPPB: Short Physical Performance Battery
WHO: World Health Organization
