# Supplementary material for: Higher hemoglobin levels are associated with better physical performance among older adults without anemia: a longitudinal analysis
Source: BMC Geriatr. 2022 Mar 21;22:233. doi: 10.1186/s12877-022-02937-4 (PMC8939094; doi:10.1186/s12877-022-02937-4)
Supplement: Supplementary file 1 — Additional file 1. [file 12877_2022_2937_MOESM1_ESM.docx]

Supplementary Table 1. Mean and standard errors of selected characteristics of older adults (≥ 60 years old) according to follow up status. SABE Study. São Paulo, Brazil, 2010.

| Characteristics | Total in baseline (n=1023) | Followed (n=545) | Lost to follow up and dead (n=478) | *p* |
| --- | --- | --- | --- | --- |
| Age | 71.56 (0.28) | 69.10 (0.32) | 74.37 (0.44) | <0.001 |
| Education (in years) | 4.85 (0.13) | 5.23 (0.18) | 4.42 (0.18) | 0.002 |
| Number of cardiometabolic conditions | 1.20 (0.03) | 1.14 (0.04) | 1.26 (0.04) | 0.043 |
| Number of other chronic conditions | 0.71 (0.02) | 0.71 (0.03) | 0.71 (0.04) | 0.921 |
| BMI (kg/m^2^) | 28.21 (0.16) | 28.50 (0.22) | 27.87 (0.24) | 0.055 |
| Hemoglobin concentration (g/dL) | 14.30 (0.03) | 14.40 (0.05) | 14.19 (0.05) | 0.005 |
| SPPB score | 8.89 (0.07) | 9.38 (0.09) | 8.34 (0.11) | <0.001 |

SE= Standard Error; 95% CI = 95% Confidence Interval; SPPB= Short Physical Performance Battery; BMI= body mass index
